# An outreach collaborative model for early identification and treatment of mental disorder in Danish workplaces

**DOI:** 10.1186/s12888-019-2027-5

**Published:** 2019-01-24

**Authors:** Helle Østermark Sørensen, Jan B. Valentin, Malene Krogsgaard Bording, Jens Ivar Larsen, Anelia Larsen, Øyvind Omland

**Affiliations:** 1 Unit for Psychiatric Research, Psychiatry, Aalborg University Hospital, Mølleparkvej 10, 9000 Aalborg, Denmark; 2 Clinic for Occupational Medicine, Aalborg University Hospital, Havrevangen 1, 9000 Aalborg, Denmark

**Keywords:** Early identification, Outreach collaborative model, Depression, Anxiety, Workplace, Unmet treatment need, Longitudinal study

## Abstract

**Background:**

Depression and anxiety are prevalent mental disorders among the working population with potentially high personal and financial cost. The overall aim of this study was to test the applicability of an outreach collaborative model for early identification and treatment of clinical and sub-clinical mental disorders among Danish employees. This applicability was examined by I) investigating the fractions of identified and treated clinical and subclinical cases, II) describing the distribution and characteristics of cases identified and III) investigating the effect of allocated treatment.

**Methods:**

A longitudinal study design with four assessments (T0-T3) over 16 months was applied. Self-reporting questionnaires probing for psychopathology were distributed to all employees in six consecutively enrolled companies at the four time points. Employees meeting the screening criteria at T1 were assessed diagnostically. Subjects diagnosed with a clinical mental disorder were allocated to outpatient psychiatric treatment, and subjects with subclinical conditions were allocated to preventive cognitive behavioural therapy. Follow-up was conducted 6 and 12 months after initiation of treatment. We used chi-squared test and F-test to compare the different groups on baseline characteristics and mixed effects linear regression to analyse the treatment effects.

**Results:**

Forty (6.8%) of the 586 responders at T1 were diagnosed with a clinical mental disorder and referred to outpatient psychiatric treatment. Thirty-three (5.6%) were affected by a subclinical condition and referred to preventive treatment. Nearly two-thirds (63%) of the employees diagnosed with a clinical condition had never received treatment before. Symptom severity decreased significantly for both treated groups until follow-up. When compared to a composed control group, subclinical cases displayed a more rapid initial significant symptomatic decrease on the global symptom scale (coefs = − 0.914, 95% CI [− 1.754, − 0,075]) and anxiety sub-scale (coefs = − 1.043, 95% CI [− 2.021, − 0.066]). This did not apply to the clinical cases as no significant difference in change were identified.

**Conclusions:**

The outreach collaborative model demonstrated an applicability to identify both clinical and subclinical cases, among these a high number of employees with an unmet need for treatment. We found evidence of a positive initial effect on symptomatology from the allocated preventive treatment among the subclinical cases, but not for clinical cases.

**Trial registration:**

Retrospectively registered at December 18, 2018 at clinicaltrials.gov, identifier: NCT03786328.

## Background

Depression and anxiety are the most prevalent mental disorders among the working population [1–3]. The conditions have potentially high personal and financial costs given their association with long-term and recurrent sickness absence, at-work performance deficit, early retirement, decreased social function, low job satisfaction and impaired quality of life [2, 4–11]. The total cost of work-related depression in the European Union was estimated to approximately €620 billion in 1 year covering productivity loss, treatment costs and disability benefit payments [12, 13].

In addition to clinical conditions, many employees are affected by symptoms of depression and anxiety at a subclinical level which may be socially inhibiting for the individual as well as negatively impacting job satisfaction, work productivity and attendance [1, 10, 14]. Untreated and not early identified, these subclinical cases can lead to actual mental disorder [15, 16].

In both the general population and the working population, evidence has demonstrated a high level of unmet need for mental health care and treatment [17–19]. The unmet needs are partly explained by diagnostic difficulties and inadequate treatment in general practice, and partly by the fact that many individuals affected do not seek professional help [20, 21].

In light of the high costs and unmet need for treatment, prevention and early detection of mental disorders among the working population ought to be a public health priority [22]. Evidence suggests that much could be gained by investing in preventive initiatives [12, 23].

Research studies within occupational mental health have mainly tested workplace-initiated stress management programmes or other universal prevention programmes using work-related outcome measures like sickness absence, work productivity and cost-effectiveness [22, 24]. Secondary prevention and early intervention programmes targeting mental health problems directly by using clinical standardised measures of depression or anxiety are limited, or suffer from limitations such as weak control conditions, short-term follow-up and lack of diagnostic assessment [22, 25–27]. A valid study by Wang et al. [28] tested a two-stage screening and outreach care management programme among American workers with clinical depression in a randomized controlled trial. Employees receiving the intervention improved significantly on both clinical and job-related outcomes at 12-month follow-up compared to employees receiving usual care. Thus, this study points at a positive return of investment from outreach and enhanced treatment programmes. However, only employees with symptom severity corresponding to at least a moderate depression were eligible for randomization.

When targeting workplace mental health problems, it is recommended to apply a collaborative approach between workplaces and mental health specialists in order to minimize the risk of misclassification and inadequate treatment [25, 29].

For this study, we therefore developed an outreach secondary prevention model to be tested in collaboration between the psychiatric treatment system and workplaces in the North Denmark Region.

The overall aim of the study was to investigate the applicability of an outreach collaborative model for early identification and treatment of clinical and sub-clinical cases of mental disorder among a Danish working population. The investigation had the following objectives:

I) To investigate the applicability of the early identification model, measured as fractions of identified clinical and subclinical cases, fraction of cases who accepted treatment and fraction of cases who completed treatment
II) To describe the distribution and characteristic of clinical and subclinical cases identified by the early identification model
III) To investigate the effect of allocated treatment on symptomatology for clinical and subclinical cases

## Methods

### Study design

**Fig. 1:**
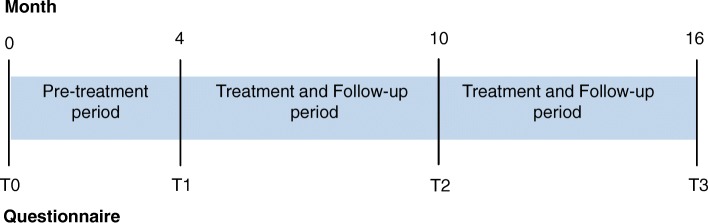
Study time line

The study applied a longitudinal naturalistic design with four points of assessment (T0-T3) during a period of 16 months for each participating company. The time from T0 to T1 (4 months) constituted a pre-treatment period and was incorporated in the design as a control period for comparison. After the second assessment (T1), screening and diagnostic assessment were initiated. Identified cases were afterwards allocated to treatment. Follow-up assessments were conducted after 6 months (T2) and 12 months (T3) for all employees. The longitudinal study design and the four assessments are illustrated in Fig. 1. The assessments used self-reporting questionnaires probing for psychopathology. All questionnaires were mailed to the employees’ private postal addresses at the four points of assessment (T0-T3) followed by a reminder after 2 weeks in case of no response.

### Participants

In order to demonstrate the model’s applicability in a representative sample, we decided to enrol six medium-large companies with a minimum of 100 employees each. Companies with more than 300 employees were not addressed due to the limitations of the available treatment capacity. After approaching 24 companies from diverse fields within both the public and private sector, six companies agreed to participate. These companies were consecutively enrolled from June 2007 to August 2013. The primary reasons for declining participation were lack of resources or time.

### Measures

The questionnaires used the Symptom Check List 90 Revised (SCL90-R) [30] as basis for identification of clinical and subclinical cases of mental disorders and follow-up to treatment. Questionnaires also collected data on demography, different work-related characteristics and job-satisfaction.

The SCL-90-R is a psychiatric self-report inventory with high reliability and validity to measure overall psychological distress and to detect changes [31–33]. In each of the 90 scale items, the responders are to assess the presence of a specific symptom on a 5-point Likert scale from 0 = “not at all” to 4 = “very much”. The quotient of the 90 items expresses the overall level of psychological distress, referred to as the Global Severity Index (GSI). The GSI-score ranges from 0 to 4 and the higher the score, the higher the level of psychological distress. The instrument also comprises nine subscales, including the depression (DEP) and the anxiety (ANX) subscales that have been established as being suitable instruments for proxies of depression and anxiety [34].

The mean GSI-level in the Danish general population has been set to 0.45 (SD: 0.43) [14], and in outpatient populations to range from 1.16 (SD: 0.49) to 1.70 (SD: 0.47) [35]. No Danish studies have published data on average distress levels for subclinical samples. A Swiss study reports mean GSI-levels for subclinical depression of 0.64 and subclinical anxiety of 0.63 [36], and German studies report mean GSI-level for primary care populations from 0.57 (SD: 0.49) to 0.89 (SD: 0.55) [37–39].

Rooted in the above GSI-levels, combined with the recommendations from the SCL90-R manual, we applied the following screening criteria for identification of clinical and subclinical cases of mental disorder [1, 30–32]:

i) GSI score ≥ 0.63 *or*
ii) values of ≥0.63 in two or more subscales *or*
iii) values of ≥0.63 in the DEP subscale

The following criteria were based on raw-scores of the SCL90-R.

### Procedure

Participants meeting the screening criteria at the second assessment (T1) were invited to a diagnostic assessment in order to determine the presence of a mental disorder. The diagnostic interviews were conducted by medical doctors in Aalborg Psychiatric Hospital, all trained and experienced users of the diagnostic instruments utilized. The presence of a mental disorder was initially determined by use of Present State Examination (PSE) [40]. Participants assessed with a state of anxiety or depression were afterwards rated on the Hamilton Depression Scale (HAM-D) [41] and/or Hamilton Anxiety Scale (HAM-A) to assess the severity of the condition [42]. Depending on the outcome of the diagnostic interview, participants were offered two different courses of treatment:

#### Treatment course I: Psychiatric treatment for clinical cases

Individuals diagnosed with a mental disorder according to the PSE, and with HAM-D score ≥ 18 or HAM-A score ≥ 20, were referred to psychiatric treatment in an outpatient specialized clinic for affective disorders in Aalborg Psychiatric Hospital. The treatment followed standard treatment procedures for psychiatric outpatients with diagnoses of depression and anxiety. Treatment was performed by trained clinicians (psychiatrists, psychologists and nurses) employed at the Clinic for Affective Disorders. The course of treatment included medical consultations as well as psychotherapeutic sessions and continued until remission was achieved.

#### Treatment course II: Preventive treatment for subclinical cases

Individuals assessed with subclinical conditions of depression or anxiety, defined as a HAM-D score of 13–17 or a HAM-A score of 15–19, were offered preventive treatment. This course of treatment consisted of eight sessions of cognitive behavioural therapy (CBT) conducted by clinical psychologists at Aalborg Psychiatric Hospital. During the sessions, the therapeutic focus was on stress-reduction and resilience.

### Statistical analysis

Initially, the primary analysis was carried out by counting the number of clinical and subclinical cases identified from the early identification procedure, as well as number of cases who accepted treatment and number of cases who completed treatment.

Secondly, we performed a descriptive analysis with the study population allocated into four groups: clinical cases, subclinical cases, untreated cases and healthy subjects. The group of untreated cases consisted of employees, who were allocated to treatment but declined the treatment offer. The group of healthy subjects consisted of all other responders, including employees with scores below cut-off point, as well as employees above cut-off points who turned out ‘false positives’ when assessed diagnostically.

The groups were compared at baseline (T1) on demography, work-related characteristics and the three outcome measures GSI, DEP and ANX. Ordinal variables were compared using chi-squared tests, and variables of continuous nature were compared using F-tests. Additionally, the same comparisons were performed between treated participants who completed the follow-up at T3 with participants who did not.

To analyse the effect of both treatments, we used mixed effects linear regression using random intercept and with participants nested inside the companies. The outcome measures were included separately as dependent variables, while the time variable (measured in years), gender, age at baseline as well as a group variable were added as covariates.

The regression analysis compared clinical and subclinical cases during the follow-up period (T1-T3) with a composed control group consisting of two subgroups. The first subgroup consisted of the untreated cases, and the second subgroup included the treated cases pre-diagnostically (T0-T1). Hence, point of origin was specified to T1 for the first subgroup and T0 for the second subgroup. To reduce the effects of regression to the mean, cases in the latter subgroup were excluded if not meeting the screening criteria at T0.

A second order time covariate was added in the regression as well as an interaction term for each of the first and second order time variables, since the differences of the initial slopes and difference in change of slopes between the groups were of interest. To investigate the effects of seasonal variations, the regressions were conducted twice with and without a variable indicating in which quarter the participant entered the period under investigation. Quantile-quantile plots were used for visual inspection of the normality assumptions.

A *p*-value < 0.05 was considered statistically significant. All analyses were carried out in Stata 13 [43].

### Ethics

Information about the study was first provided to the workplace management and the health and safety representatives and subsequently to all employees at joint open meetings.

It was emphasised that the study was voluntary at all levels. Written participant information accompanied each questionnaire. The return of the questionnaires was considered as acceptance of participation in the questionnaire part. Written informed consent was obtained from all participants involved in diagnostic interview and treatment. The employees were guaranteed complete anonymity in relation to their workplace and company management. The study was approved by the Danish Data Protection Agency (j. 2008-58-0028). The study was presented to the Danish Scientific Ethics Committee (N-20070016), but the need for approval was waived due to the nature of the study.

## Results

### Questionnaire response

Three public and three private corporations with an average number of 178 employees (range 130–240) at the time of inclusion were enrolled. All individuals employed at each point of time received the questionnaires. The questionnaire population were, therefore, slightly different at each time-point, due to staff turnover.

At baseline (T1), a total of 586 (54.9%) employees of the study population (*n* = 1068) returned the questionnaire and were assessed according to the screening criteria. Non-responders thereby comprised 482 employees (45.1%). There was a higher proportion of women among the responders (41.1%) than among non-responders (31.7%) at baseline. The average age for the responders were 45 years (min: 21, max: 67). Response rates were higher than non-response rates in company 1, 2 and 3 (above 50%), and lower in company 4, 5 and 6 (below 50%).

All response rates for each company at the four time points are listed in Table 1.

**Table 1:** Response rates for all companies at all time points (T0-T3)

Company	Recipients N	Response N (%)	Recipients N	Response N (%)	Recipients N	Response N (%)	Recipients N	Response N (%)
	T0: pre-treatment assessment		T1: Screening, diagnostic assessment and initiation of treatment		T2: 6-month follow-up		T3: 12-month follow-up	
1. Public service	163	107 (65.6)	167	101 (60.5)	170	96 (56.5)	180	100 (55.6)
2. Public service	166	100 (60.2)	167	94 (56.3)	170	106 (62.4)	160	97 (60.6)
3. Private, financial	251	181 (72.1)	240	161 (67.1)	247	161 (65.2)	246	166 (67.5)
4. Private, manufacturing	148	74 (50.0)	162	71 (43.8)	165	71 (43.0)	156	69 (44.2)
5. Private, manufacturing	125	59 (47.2)	130	55 (42.3)	135	50 (37.0)	139	59 (42.4)
6. Public, education	198	88 (44.4)	202	92 (45.5)	185	82 (44.3)	182	72 (39.6)
Total	1051	609 (57.9)	1068	586 (54.9)	1072	566 (52.8)	1063	563 (53.0)

### Identified cases

Altogether 160 individuals met the screening criteria and were invited to a diagnostic assessment and 130 accepted the invitation. Based on the diagnostic assessment with the PSE, 73 were classified with either a clinical or subclinical condition of mental disorder and assigned to either psychiatric treatment (*n* = 40) or preventive treatment (*n* = 33). Fifteen declined the treatment offer or did not receive treatment as part of the study, either due to other available treatment options, spontaneous recovery or non-attendance. Thus, 38 individuals classified with a clinical mental disorder received psychiatric standard treatment with a mean treatment period of 369 days (sd = 314; min = 52, max = 1162), and 20 individuals identified with a subclinical condition received preventive CBT treatment with a mean treatment period of 103 days (sd = 81; min = 31, max = 343).

The cases identified correspond to a prevalence of 6.8% for a clinical condition of mental disorder, and 5.6% for a subclinical condition.

**Fig. 2:**
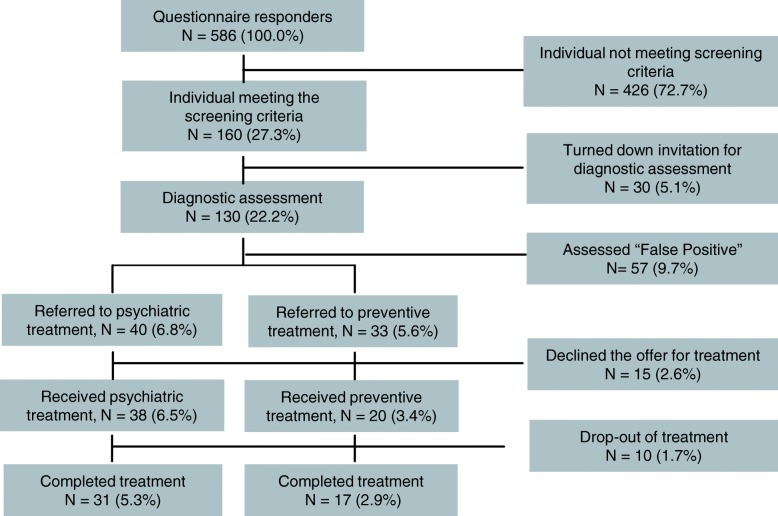
Screening and identification of clinical and subclinical cases of mental disorders

Figure 2 illustrates the procedure of the identification of clinical and subclinical cases.

### Baseline characteristics

Baseline characteristics for the clinical, subclinical and untreated cases as well as healthy subjects are listed in Table 2. We observed a statistically significant higher proportion of women in the case groups than among healthy subjects. Moreover, number of sick days during the latest month, presence of physical illness and treatment with psychotropic drugs were significantly higher among all case groups than among healthy subjects. Job-satisfaction was significantly lower in all case groups than among healthy subjects.

**Table 2:** Sociodemographic characteristics for the study population at initiation of treatment (T1), N (%)

	Clinical cases (*n* = 38)	Subclinical cases (*n* = 20)	Untreated cases (*n* = 15)	Healthy subjects (*n* = 513)
Sex				
Female	22 (57.9)	7 (35.0)	11 (73.3)	201 (39.2)
Male	16 (42.1)	13 (65.0)	4 (26.7)	312 (60.8)
Age				
Years (mean, sd)	43.6 (9.8)	44.8 (9.3)	46.9 (10.3)	45.3 (9.9)
Marital status				
Married/live together	27 (71.1)	15 (75.0)	14 (93.3)	432 (84.2)
Single	11 (28.9)	5 (25.0)	1 (7.1)	81 (15.8)
Company				
1: public service	0 (0.0)	6 (30.0)	2 (13.3)	92 (17.9)
2: public service	7 (18.4)	7 (35.0)	1 (6.7)	79 (15.4)
3: private, financial	11 (28.9)	3 (15.0)	6 (40.0)	154 (30.0)
4: private, manufacturing	6 (15.8)	2 (10.0)	1 (6.7)	62 (12.1)
5: private, manufacturing	5 (13.2)	0 (0.0)	2 (13.3)	48 (9.4)
6: public, education	9 (23.7)	2 (10.0)	3 (20.0)	78 (15.2)
Working hour system				
Day work	24 (63.2)	17 (85.0)	8 (53.3)	359 (70.0)
Shift work	5 (13.1)	1 (5.0)	3 (20.0)	67 (13.1)
24-h shift	1 (2.6)	1 (5.0)	0 (0.0)	28 (5.5)
Other	8 (21.1)	1 (5.0)	4 (26.7)	59 (11.5)
Field of work				
Administration/management	9 (23.7)	5 (25.0)	6 (40.0)	158 (30.8)
Internal logistics and service	13 (34.2)	9 (45.0)	3 (20.0)	136 (26.5)
Teaching	4 (10.5)	2 (10.0)	2 (13.3)	55 (10.7)
Customer service	5 (13.2)	3 (15.0)	0 (0.0)	71 (13.8)
Production	7 (18.4)	0 (0.0)	3 (20.0)	38 (7.4)
Rescue work	0 (0.0)	1 (5.0)	1 (6.7)	50 (9.7)
Other	0 (0.0)	0 (0.0)	0 (0.0)	2 (0.4)
N/A				3 (0.6)
Job-satisfaction (mean, sd)	7.3 (2.2)	6.7 (2.2)	7.8 (2.5)	8.6 (1.8)
Length of service				
Years (mean, sd)	16.7 (10.6)	17.7 (11.4)	20.9 (12.8)	19.3 (11.6)
Work hours per week				
Hours (mean, sd)	35.4 (8.0)	36.6 (4.4)	36.1 (3.0)	37.1 (4.6)
Overtime latest month				
Hours (mean, sd)	10.7 (30.9)	4.7 (9.4)	1.9 (4.2)	5.6 (14.7)
Sick days latest month				
Days (mean, sd)	4.3 (9.0)	0.2 (0.6)	1.8 (2.3)	0.6 (2.4)
Physical illness				
Yes	13 (34.2)	9 (45.0)	7 (46.7)	120 (23.4)
No	25 (65.8)	11 (55.0)	8 (53.3)	393 (76.6)
In treatment with psychotropic drugs				
Yes	7 (18.4)	2 (10.0)	3 (20.0)	16 (3.1)
No	31 (81.6)	18 (90.0)	12 (80.0)	497 (96.9)
Season for screening				
1. quarter (Jan-Mar)	11 (28.9)	3 (15.0)	6 (40.0)	148 (28.8)
2. quarter (Apr-Jun)	1 (2.6)	0 (0.0)	0 (0.0)	5 (1.0)
3. quarter (Jul-Sep)	22 (57.9)	17 (85.0)	6 (40.0)	300 (58.5)
4. quarter (Oct-Dec)	4 (10.5)	0 (0.0)	3 (20.0)	60 (11.7)

*Mean* mean score, *sd* standard deviation

No statistically significant differences on baseline characteristics were found when comparing responders with non-responders at follow-up.

### Unmet need for treatment

Of the 40 employees identified with a clinical mental condition, 33 were diagnosed with a depressive disorder, 1 with bipolar affective disorder, 1 with anxiety disorder, 2 with adjustment disorder and 1 with personality disorder. The remaining 2 individuals were not definitively diagnosed as they rejected the treatment offer. Prior to the initiation of treatment, only 14 (35.0%) of the 40 individuals identified with a clinical mental condition had received treatment before (anti-depressive medication and/or psychological treatment). Thus, 26 (65.0%) had never pursued or received any treatment earlier in life, corresponding to a prevalence of 4.1% of the study sample. Seven (21.2%) of the employees identified with a subclinical condition had previously received treatment for their mental health problems, and 23 (69.7%) had never pursued or received treatment, corresponding to a prevalence of 3.9% of the study sample.

### Treatment effect

In Table 3, the symptom scores (GSI, DEP and ANX) at the four measurement points are listed for all groups. Scores decreased statistically significant from initiation of treatment (T1) to 12-month follow-up (T3) for all treated participants regardless of treatment, with the exception of the DEP-subscale for subclinical cases merely demonstrating a trend-significant change. Untreated cases also demonstrated a statistically significant reduction on the GSI and ANX scores. No significant reduction in symptom severity for healthy subjects was observed.

**Table 3:** Changes on symptomatology during the study period (T0-T3), mean (sd)

	Clinical cases					
	T0 (*n* = 32)	T1 (*n* = 38)	T2 (*n* = 26)	T3 (*n* = 28)	Co-efficient (CI), treatment period (T1-T3)	*p*-value
Global Severity Index (GSI)	1.07 (0.49)	1.15 (0.44)	0.94 (0.56)	0.75 (0.67)	−0.385 (− 0.576, − 0.194)	< 0.001
Subscale – depression (DEP)	1.60 (0.79)	1.74 (0.62)	1.43 (0.88)	1.18 (0.93)	−0.574 (− 0.849, − 0.244)	0.004
Subscale – anxiety (ANX)	0.89 (0.72)	0.98 (0.73)	0.78 (0.67)	0.53 (0.73)	−0.377 (− 0.561, − 0.193)	< 0.001
	Subclinical cases					
	T0 (*n* = 17)	T1 (*n* = 20)	T2 (*n* = 14)	T3 (*n* = 12)	Co-efficient (CI), treatment period (T1-T3)	*p*-value
Global Severity Index (GSI)	0.75 (0.48)	0.74 (0.33)	0.46 (0.27)	0.53 (0.36)	−0.236 (− 0.457, − 0.016)	0.036
Subscale – depression (DEP)	1.01 (0.75)	1.07 (0.67)	0.75 (0.56)	0.75 (0.55)	−0.363 (− 0.748, 0.022)	0.064
Subscale – anxiety (ANX)	0.58 (0.55)	0.52 (0.37)	0.21 (0.21)	0.36 (0.40)	−0.220 (− 0.431, − 0.009)	0.041
	Untreated cases					
	T0 (*n* = 11)	T1 (*n* = 15)	T2 (*n* = 8)	T3 (*n* = 9)	Co-efficient (CI), treatment period (T1-T3)	*p*-value
Global Severity Index (GSI)	0.73 (0.56)	0.76 (0.39)	0.41 (0.30)	0.49 (0.22)	−0.333 (− 0.581, − 0.085)	0.009
Subscale – depression (DEP)	1.06 (0.77)	1.13 (0.57)	0.75 (0.59)	0.88 (0.41)	−0.348 (− 0.725, 0.028)	0.070
Subscale – anxiety (ANX)	0.53 (0.51)	0.64 (0.47)	0.29 (0.35)	0.22 (0.16)	−0.433 (− 0.708, − 0.159)	0.002
	Healthy Subjects					
	T0 (*n* = 419)	T1 (*n* = 512)	T2 (*n* = 389)	T3 (*n* = 370)	Co-efficient (CI), treatment period (T1-T3)	*p*-value
Global Severity Index (GSI)	0.25 (0.27)	0.21 (0.25)	0.19 (0.21)	0.20 (0.25)	0.007 (−0.012, 0.026)	0.472
Subscale – depression (DEP)	0.33 (0.42)	0.27 (0.38)	0.27 (0.35)	0.28 (0.39)	0.027 (−0.005, 0.058)	0.096
Subscale – anxiety (ANX)	0.17 (0.27)	0.14 (0.28)	0.13 (0.24)	0.13 (0.23)	0.001 (−0.022, 0.023)	0.957

*Mean* mean scale score for each group, *sd* standard deviation, Co-efficients are reported with confidence intervals (CI)

There was a notable amount of missing responses in all groups at 12-month follow-up, *n* = 10 (26.3%) among clinical cases, *n* = 8 (40.0%) among subclinical cases, *n* = 6 (40.0%) among un-treated cases and *n* = 142 (27.7%) among healthy subjects.

There were no statistically significant differences on outcome measures at baseline when comparing responders with non-responders at follow-up.

In Table 4, the results from the main regression analysis including seasonal variations are displayed. Here, we compared the difference in change over time between the treated case groups and the control group by investigating initial decline and overall change of slopes. No significant difference in change was detected in any outcome measures for clinical cases. For subclinical cases, the initial slope and the change of slope differed statistically on the GSI and ANX scales, but not on the DEP-scale.

**Table 4:** Difference in changes of symptomatology over time. Comparing treated cases and controls

	Initial difference of slope per year^a^ (first order time covariate)				Change of difference of slope over time per half year (second order time covariate)			
	Coefs	CI		*p*-value	Coefs	CI		*p*-value
Clinical cases								
Global Severity Index (GSI)	− 0.254	−1.064	0.556	0.540	0.161	−0.701	1.023	0.714
Depression sub-scale (DEP)	−0.433	−1.698	0.831	0.502	0.238	−1.108	1.583	0.730
Anxiety sub-scale (ANX)	−0.294	−1.168	0.581	0.510	0.256	−0.676	1.188	0.591
	Coefs	CI		*p*-value	Coefs	CI		*p*-value
Subclinical cases								
Global Severity Index (GSI)	−0.914	−1.754	−0.075	0.033	0.945	0.065	1.825	0.035
Depression sub-scale (DEP)	−1.092	−2.399	0.215	0.101	1.054	−0.316	2.424	0.132
Anxiety sub-scale (ANX)	−1.043	−2.021	−0.066	0.036	1.157	0.132	2.182	0.027

Analyses are adjusted for age at T1, gender and seasonal variation and nested in companies

*Coefs* coefficient, *CI* confidence interval

^a^Negative value = relative decline

## Discussion

The study investigated the applicability of an outreach collaborative model for early identification and treatment of clinical and subclinical conditions of mental disorder among a sample of Danish employees. The model identified prevalence’s for clinical and subclinical disorders similar to other studies and found evidence pointing at a noticeable effect of early treatment for subclinical cases measured on overall psychopathology. An unpredicted finding was, however, the identification of a high amount of unmet need for treatment for both subclinical and clinical cases. Approximately 2/3 of the employees diagnosed with a clinical diagnosis had never received treatment for their mental health problems before.

Previous studies found that half of the individuals with treatment needs were untreated or received inadequate treatment [18, 44]. Our study detects a considerable higher proportion of unmet need for treatment, which points at a pronounced need for initiatives for early detection and treatment.

The prevalence estimates for clinical and subclinical cases of mental disorder identified in this study correspond with earlier findings in working populations [1–3, 14]. The reliability of these prevalence estimates is enhanced by the fact that all subjects meeting the screening criteria were assessed diagnostically by well-trained psychiatrists. However, there was a large proportion of non-responders, which might have biased our prevalence estimates.

In regard to treatment effect, we observed a statistically significant improvement in symptom severity among both clinical and subclinical cases, equivalent to the decline in depressive symptoms over a 12-month period in the study by Wang et al. [28]. The untreated cases also demonstrated a significant reduction in symptom severity. The reason for this is unknown, but it could be an effect of regression to the mean. However, we are aware that some of these cases declined the study offer for treatment due to other available treatment options.

When comparing the symptomatic changes for the treated groups with a composed control group, the treatment effect were less evident. Among the clinical cases, the symptomatic changes followed nearly the same course as controls. Subclinical cases displayed a more rapid initial decline in symptoms compared to controls. A possible explanation might be that subclinical cases were identified at a point of time where their mental health problems were still of minor severity. Therefore, they probably responded more rapidly to treatment than clinical cases, which entered treatment in a more severe mental health state. The rapid decline was, however, equalized and transformed to a minor relative growth after 6 months. The effect of the preventive treatment might also be connected to the type of treatment. CBT treatment was considered the best treatment option in two systematic reviews on preventive interventions for common mental disorders [23, 27].

### Limitations

The findings of this study should be considered with care. In regard to the analysis of the treatment effect, the main limitations were the lack of randomisation and the use of the companies/employees as own controls. Due to the lack of independent controls, we included the pre-treatment assessment (T0) in the design as a period for comparison. Our design therefore introduces definite possibility for misclassification, but we have no reason to believe that we have introduced a differential misclassification. However, a non-differential misclassification, tending to reduce the effect of treatment towards zero, cannot be excluded.

The lack of control group (randomization) seems to be a general limitation in workplace directed studies targeting mental health problems, which indicates difficulties in recruiting companies for the sole purpose of being controls [22]. We estimated that using the companies as own controls was the best overall design including ethical issues and real-life circumstances. We experienced, like several other longitudinal studies, a substantial reduction in participation rate. This adds to the limitations of our findings, most probably by introducing a non-differential misclassification reducing the odds for a significant finding of treatment effect.

The defined control group was composed of a diverse crowd with somewhat weaker follow-up conditions due to a shorter pre-assessment period as well as a high amount of non-responders at follow-up among untreated cases. Hence, available data were sparse at 12-month follow-up, leaving the regression line highly influenced by the data at baseline and at 4- and 6-month follow-up. Thus, the course of symptoms for the control group may have been misinterpreted. In addition, the symptomatic change among all participants might be effected by regression to the mean as well as seasonal variations, though these effects have been minimized in the analysis. Moreover, there is a risk of the results being biased by internal working conditions, work cultures or other similarities among workers in specific professions [45]. We attempted to account for this by including both private and public companies from diverse line of business and by including all employees regardless of education levels. Nonetheless, a non-differential misclassification might have been introduced tending to reduce the treatment effect to zero.

A major strength of this study was the diverse sample of Danish employees from different industries and sectors enhancing the generalisability of the findings. The study also benefitted from incorporating a long follow-up period, using validated measures for psychological distress as well as diagnostic verification of the self-reported screening results.

## Conclusion

In summary, this outreach collaborative model for early identification seems applicable for identification of both existing insufficiently treated cases as well as untreated cases of both clinical and subclinical levels of mental disorder among a diverse group of Danish employees. The models’ eligibility are further supported by the identified high level of unmet need for treatment among the study sample, which advocates for further development of such outreach collaborative models.

Although both treated clinical and subclinical cases experienced a clinically important improvement in symptomatic levels, the study did not produce any hard evidence that this was due to the treatment, primarily due to weak control conditions. Best valuable evidence was found for the efficiency of early treatment for sub-clinical cases. A long-term follow-up in the Danish national registers on occupational status and health conditions may reveal any difference between treated and untreated cases in long-term effect of the early identification and treatment.

## Abbreviations

ANX: Anxiety subscale
CBT: Cognitive Behavioural Therapy
DEP: Depression subscale
GSI: Global Severity Index
HAM-A: Hamilton Anxiety Scale
HAM-D: Hamilton Depression Scale
PSE: Present State Examination
SCL-90-R: Symptom Check List 90 Revised
